# Improving the Performance of an Electronic Nose by Wine Aroma Training to Distinguish between Drip Coffee and Canned Coffee

**DOI:** 10.3390/s150101354

**Published:** 2014-10-15

**Authors:** Kouki Fujioka, Yasuko Tomizawa, Nobuo Shimizu, Keiichi Ikeda, Yoshinobu Manome

**Affiliations:** 1 Core Research Facilities for Basic Science, The Jikei University School of Medicine, Minato-ku, Tokyo 105-8461, Japan; E-Mails: ikedak@jikei.ac.jp (K.I.); manome@jikei.ac.jp (Y.M.); 2 Department of Cardiovascular Surgery, Tokyo Women's Medical University, Shinjuku-ku, Tokyo 162-8666, Japan; E-Mail: stomizaw@hij.twmu.ac.jp; 3 Department of Data Science, The Institute of Statistical Mathematics, Tachikawa, Tokyo 190-8562, Japan; E-Mail: nobuo@ism.ac.jp

**Keywords:** electronic nose, coffee, Le Nez du Vin, wine aroma, similarity, FF-2A, objective description

## Abstract

Coffee aroma, with more than 600 components, is considered as one of the most complex food aromas. Although electronic noses have been successfully used for objective analysis and differentiation of total coffee aromas, it is difficult to use them to describe the specific features of coffee aroma (*i.e.*, the type of smell). This is because data obtained by electronic noses are generally based on electrical resistance/current and samples are distinguished by principal component analysis. In this paper, we present an electronic nose that is capable of learning the wine related aromas using the aroma kit “Le Nez du Vin,” and the potential to describe coffee aroma in a similar manner comparable to how wine experts describe wine aroma. The results of our investigation showed that the aromas of three drip coffees were more similar to those of pine and honey in the aroma kit than to the aromas of three canned coffees. Conversely, the aromas of canned coffees were more similar to the kit coffee aroma. In addition, the aromatic patterns of coffees were different from those of green tea and red wine. Although further study is required to fit the data to human olfaction, the presented method and the use of vocabularies in aroma kits promise to enhance objective discrimination and description of aromas by electronic noses.

## 1. Introduction

Coffee aroma has more than 600 components and is considered to be one of the most complex food/drink aromas [1]. Recent studies have demonstrated the difficulties of describing coffee aromas based on the components. For example, it has been observed that the interaction between furfuryl mercaptan (a major component of coffee aroma) and short-chain carboxylic acids affects human olfaction [2,3], and that the peak areas of 40 important compounds in coffee volatiles were different for different sampling times [4]. This has made it difficult to describe the features of total coffee aroma based on the components.

Cup tests are generally used for expert assessment of the qualities of coffee, including the aroma [5]. However, quality control by testers is subjective and depends on the skill of the testers [5]. Hence, only qualified testers are capable of detailed differentiation among the qualities of coffees.

To solve the problem of difficulty and subjectivity, electronic noses have been used for coffee evaluation [1]. For example, Gardner *et al.* classified several coffee aromas with a success rate of 81.1%–95.5% by analyzing the headspace of the coffee pack using an array of thin oxide sensors [6]. Rodriguez *et al.* classified several coffee groups such as healthy coffees and fermented coffees using metal-oxide gas sensors [5]. Despite the successful use of electronic noses for coffee evaluation and discrimination, it is difficult to use the technique to describe the features of coffee aroma (*i.e.*, the type of smell) directly. This is because the data obtained by electronic noses are generally in the form of electrical resistance/current and the classification of samples is based on principal component analysis.

In this study, we used the FF-2A electronic nose (Shimadzu Corporation, Kyoto, Japan) to investigate aroma samples to develop a method for objective description of the aromatic features. The special feature of the FF-2A electronic nose is its ability to calculate the similarities among the nine standard gasses [7–12].

In our previous study, we expanded the ability of electronic noses to include the description of aroma by recording the smells of wine using the aroma kit Le Nez du Vin. [13]. Le Nez du Vin contains 54 aroma solutions based on a descriptor of wine aroma and off-flavors such as apricot and tar, and is generally used for human learning of wine aromas (*i.e.*, wine expert training). For example, Tao *et al.* showed that students exhibited improved aroma discrimination ability after training by Le Nez du Vin [14]. In our previous study, the electronic nose was trained using the 51 aromas from Le Nez du Vin, and was able to discriminate a drip coffee (Colombia coffee) from instant coffee (Colombia-type coffee) based on similarity to seven aromas (coffee, mushroom, pine, honey, strawberry, musk, and caramel) [13]. In the present study, we investigated the applicability of the method to other coffees, and compared the coffees with wine and green tea using an electronic nose trained by Le Nez du Vin.

## 2. Experimental Section

### 2.1. Coffee, Green Tea, Red Wine

We investigated three drip coffees and three canned coffees. The drip coffees were brewed by two coffee shops belonging to the same chain, and the canned coffees were selected from among the blends of the same company. The similarity values of the three canned coffees were obtained from a conference proceeding [15]. The Japanese green tea in PET bottle was selected as the green tea sample, and Cabernet Sauvignon and Syrah in pack was used as the red wine sample. The details of the coffee and wine samples are given in Table 1.

### 2.2. Aroma Samples from Le Nez du Vin

To calculate the similarities to 51 aromas in Le Nez du Vin (French edition by Jean Lenoir), we used previously measured resistance values [13]. The 51 aromas: 1. Abricot (Apricot), 2. Acacia, 3. Amande amere (Almond), 4. Ananas (Pine apple), 5. Anis, 6. Aubepine (Hawthorn), 7. Banane (Banana), 8. Beurre (Butter), 9. Cacao, 10. Cannelle (Cinnamon), 11. Café (Coffee), 12. Caramel, 13. Cassis (Black currant), 14. Cerise (Cherry), 15. Champignon (Mushroom), 16. Chene (Oak), 17. Citron (lemon), 18. Civette (Chive), 19.Coing (Quince), 20. Eglantine (Wild rose), 21. Foine coupe (Hay), 22. Fougere (Fern), 23. Fraise (Strawberry), 24. Framboise (Raspberry), 25. Fume, 26. Geranium, 27. Girofle (Clove), 28. Goudron (Tar), 29. Iode (Iodine), 30. Muscat, 31. Menthe (Mint), 32. Mercaptan, 33. Miel (Honey), 34. Musc (Musk), 35. Noisette (Hazelnut), 36. Noix (Walnut), 37. Orange, 38. Pivoine (Peony), 39. Pin (Pine), 40. Poire (Pear), 41. Poivre (Pepper), 42. Poivron-vert (Green pepper), 43. Pomme (Apple), 44. Prune, 45. Rose, 46. Soufre (Sulfur), 47.Thyme, 48. Tilleul (Lime), 49. Truffe (Truffle), 50. Viollete (Violet), and 51. Vinaigre (Vinegar).

### 2.3. Sample Measurements and Analyses Using FF-2A Electronic Nose

The measurement and analysis methods for the FF-2A (Shimadzu Corporation) are described in our previous paper [7]. The coffee samples (200 μL) were put into different 2-L PET bags (Shimadzu Corporation) filled with G1 grade dry nitrogen. The contents of the bags were then allowed to equilibrate for 30 min at room temperature. Green tea (500 μL) and red wine (5 μL) were measured for comparison after equilibration times of 30 and 5 min, respectively.

With regard to the Le Nez du Vin flavor, about 5 μL samples (liquid) were collected in 2 L-PET bags filled with dry nitrogen, and the first bag was equilibrated for 30 min. Considering that some of the aroma samples were of higher or lower concentrations than required for sensing by the semiconductor sensors (maximum resistant ratio >2 or <0.6), the gas sample in the head space of the first bag was either diluted with the nitrogen in another 2-L PET bag using an appropriate ratio or concentrated with 10 or 20 μL of the liquid flavor (see Table 2), and then equilibrated. The equilibrated sample was introduced into FF-2A electronic nose and exposed to the array of 10 sensors with pure nitrogen gas in both direct and capture modes. All the samples were measured four times and the last three measurements were used for the analyses (*n* = 3).

To calculate the similarities [7], the measurement data for each of the coffee, green tea, and red wine samples was compared with the average data for the flavor samples (based on three measurements) using the Asmell2 software in the medium mode (Shimadzu Corporation). SPSS 17.0.1 (IBM, Armonk, NY, USA) and Excel 2013 (Microsoft Corporation, Redmond, WA, USA) were respectively used for the principal component analyses (PCA) without rotation and the Pearson's correlation analyses with *p*-values.

## 3. Results and Discussion

**Figure 1.**
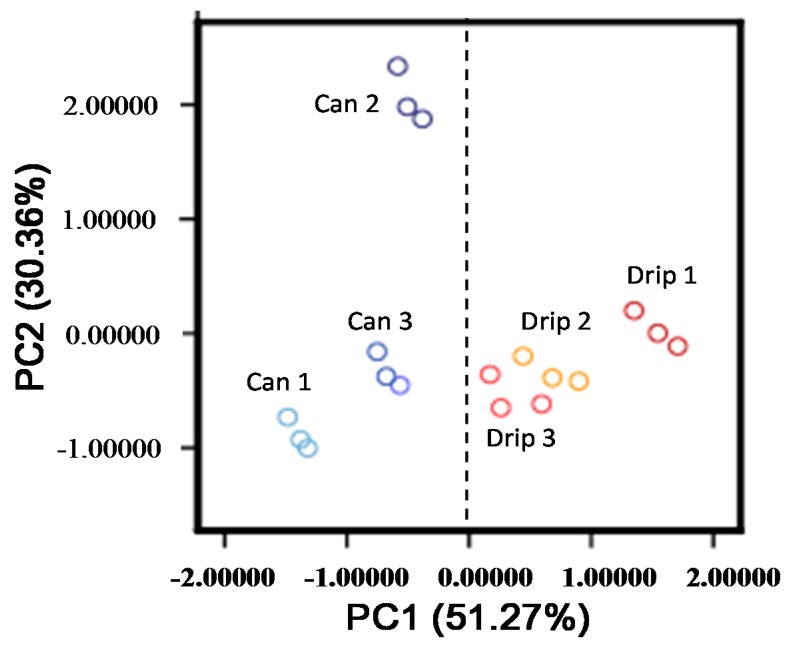
PCA of electronic resistances of drip and canned coffees (*n* = 3).

We first measured the resistances of the coffee aromas. Figure 1 shows the results of the PCA of the coffee aromas (three drip coffees and three canned coffees) using 10 sensors. Because the drip and canned coffees were divided into separate groups in PC1, the sensor sets could be used to distinguish them. However, we could not determine why Canned 2 was separated from the other samples in PC2.

**Figure 2.**
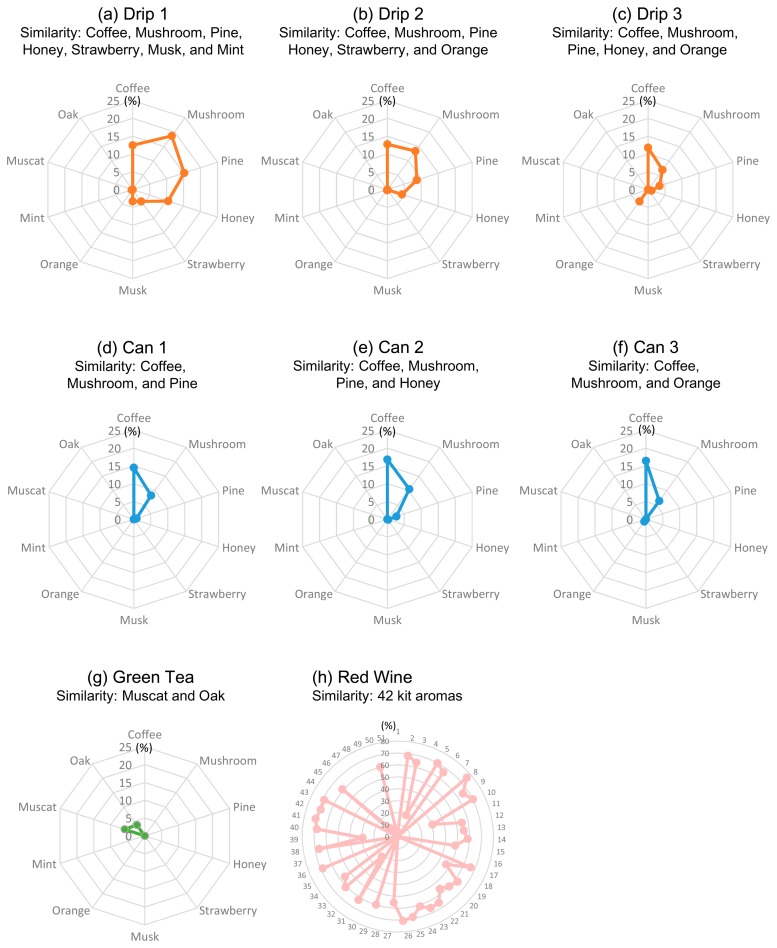
Aromatic similarity patterns of coffees (**a**–**f**), green tea (**g**), and red wine (**h**) (*n* = 3). The similarity levels are expressed in %. The loop numbers in (h) correspond to the aroma numbers presented in the experimental section. The similarity values of Canned 1–3 (**d**–**f**) were obtained from a conference proceeding [15].

Next, we investigated the aromatic features of the coffees based on similarity to 51 aromas in the Le Nez du Vin aroma kit. The similarity values compared to eight kit aromas (coffee, mushroom, pine, honey, strawberry, musk, orange, and mint) exceeded zero (Figure 2). All the drip coffees exhibited aromatic similarity to kit coffee, mushroom, pine, and honey. However, Canned 3 did not exhibit similarity to pine and honey, and Canned 1 did not exhibit similarity to honey. Nevertheless, all the canned coffees exhibited aromatic similarity to kit coffee and mushroom.

Furthermore, compared to the canned coffees, the drip coffees were similar to a wide variety of kit aromas. Drip 1, 2, and 3 were similar to seven, six, and five kit aromas, respectively, whereas Canned 1, 2, and 3 were similar to three, four, and three aromas, respectively (Figure 2). In addition, we compared the aromatic expressions of green tea and red wine with those of coffee (Figure 2). Whereas coffee exhibited eight different aromas, green tea expressed two aromas (muscat and oak) only, and red wine expressed 42 aromas. This demonstrated the differences among the aromatic patterns of different drinks, and the overall results suggested the ability of electronic noses to distinguish drinks' aromas.

**Figure 3.**
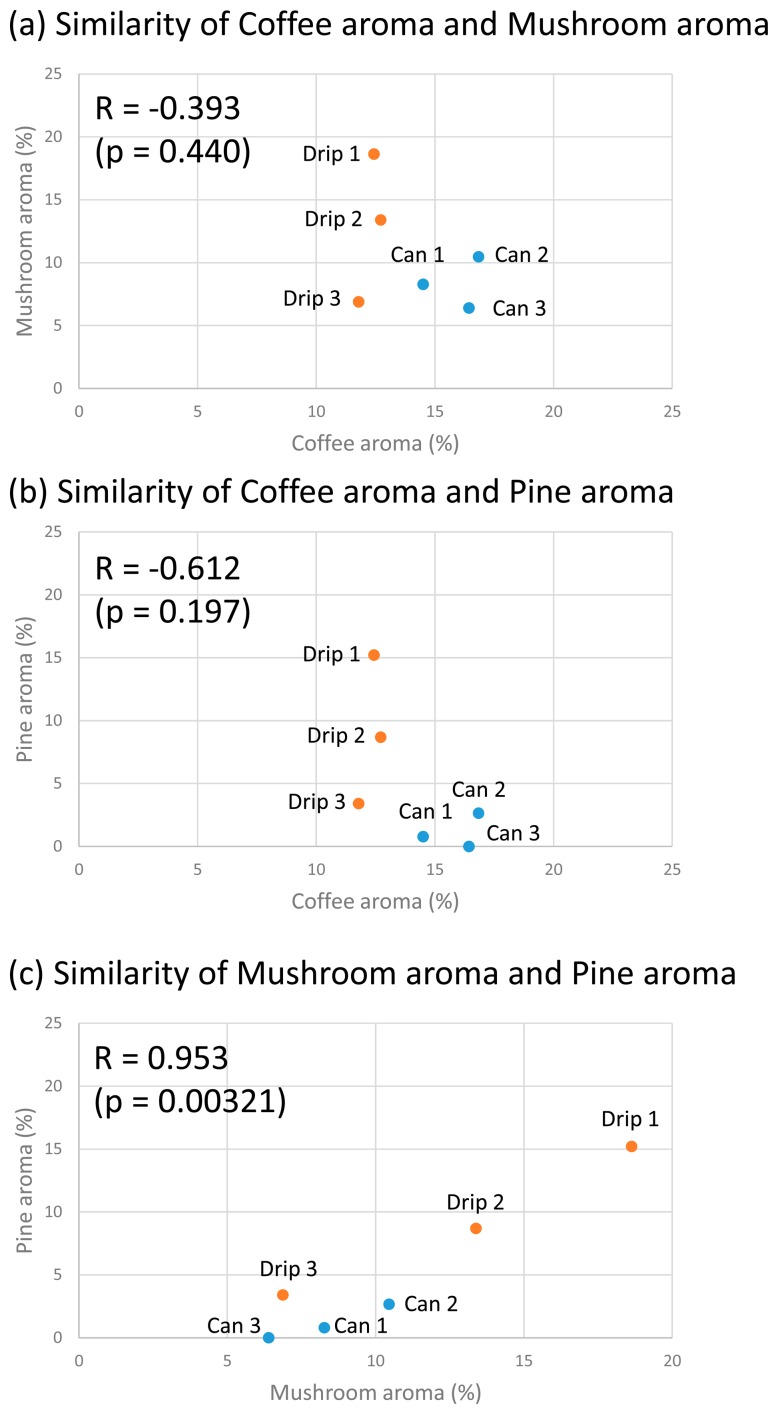
Aromatic similarity plot of drip and canned coffees against kit coffee, mushroom, and pine (average, *n* = 3). The similarity values of Canned 1–3 were obtained from a conference proceeding [15].

We also examined the relationship among the key coffee aromas, namely, kit coffee, mushroom, and pine (Figure 3). The correlation coefficient between kit coffee and mushroom (R = −0.393, *p* = 0.440) and between kit coffee and pine (R = −0.612, *p* = 0.197) were not up to the significance level of 5%. Conversely, there was a high correlation between mushroom and pine (R = 0.953, *p* = 0.00321). This suggests that these aroma axes belong to a similar category in coffee aroma description by the electronic nose or that mushroom-like and pine-like aroma present in the coffee, resulting in the correlation. Interestingly, compared to the canned coffees, the drip coffee was less aromatically similar to the kit coffee aroma and more similar to pine.

It is thus evident that an FF-2A electronic nose trained by wine aromas can be used to distinguish between drip and canned coffees, and has the potential for objective description of the aromatic features of coffee. The similarity data are easier to understand compared to the results of PCA (Figure 1) and afford more aromatic information.

It is worthwhile to discuss the meanings of the detected aromas, although further study is necessary to fit the data to human olfaction for accuracy. All the coffee aromas recognized by the FF-2A electronic nose and their attribution in the wine and coffee wheels are given in Table 3.

Firstly, the similarity to the kit coffee aroma suggests that the electronic nose successfully recognized the volatiles of the coffees as being coffee. It also indicates a burned flavor considering that the descriptor of coffee flavor in a wine aroma wheel indicates a burned (or more broadly, woody) feeling [16]. Interestingly, the drip coffees were less aromatically similar to the kit coffee aroma (Figures 2 and 3). This suggests that the complexity and interaction of the drip coffee aroma components are less similar to those of the kit coffee aroma, or that the coffee aroma components such as furfuryl mercaptan were of lower concentration relatively.

Secondly, the mushroom aroma indicates an earthy feeling in the wine aroma wheel [16] or groundy feeling in the coffee flavor wheel of the Specialty Coffee Association of America [17]. The mushroom aroma may also indicate a Koku taste/flavor (mouthfulness and continuity of the flavor); 1-octen-3-ol, which is the main component of mushroom aroma, has been found to contribute to the Koku taste in seasoning soy sauce [18]. In either case, it is necessary to compare the results of the electronic nose with those of human olfaction. Because the range of values in the mushroom aroma axis is wide (drip: 6.9%–18.7%, canned: 6.4%–10.5%; Figures 2 and 3), we could not determine the overall tendency from the obtained results.

Moreover, pine, honey, strawberry, and mint aromas respectively indicate turpeny (or more broadly, resinous), caramerized/syrup-like (caramelized/caramelly), berry (fruity), and fresh (vegetative) feelings in the aroma wheels [16,17]. The greater aromatic similarities of the drip coffees to pine and honey compared to the canned coffees (Figures 2 and 3) may be attributed to the difference between drip and canned coffees. Strawberry (Drip 1 and 2) and Mint (Drip 1) aromas were only detected in the drip coffees.

Other aromas orange (Canned 3, Drip 2, and 3) and musk (Drip 1) are not described in the two considered wheels [16,17]. Orange aroma may belong to Citrus in both wheels, indicating fruity. On the other hand, the real musk aroma was a fascinating animalistic scent [19] and is known to decrease the cortisol level, indicating attenuation of stress, and to change the testosterone level (up-regulated in female; down-regulated in male) [20]. If we recognize the musk-like aroma in the coffee, these hormones may be affected unconsciously.

There is the question of whether the aromatic similarities detected by the electronic nose are related to those detected by human olfaction. A previous study showed that the aroma of Colombia coffee (medium roast) is expressed by 13 descriptors, namely, smoky, roasted, fatty, earthy, curry, burnt, pungent, fruity, floral, caramel, sulphurous, toasted bread, and coffee [21]. Compared to the aroma descriptors determined from the data for Drip 1 (Colombia coffee, medium roast) in this study, including the broader descriptors in the aroma wheel classification, there are five agreements, namely, earthy (mushroom according to the electronic nose), burnt (coffee), fruity (strawberry), caramel (honey), and coffee (coffee). The accuracy rate relative to human descriptors is thus 5 (coffee ×2, mushroom, strawberry, and honey)/13 = 38.5%. Considering that the number of ways in which four aromas can be selected from the total of 51 kit aromas is _51_C_4_ = 249,900, the accuracy rate may be higher than expected.

There is, however, room for future improvement. Firstly, the similarity data requires better fitting to human olfaction because the sensitivity of the human olfactory sense differs from that of the FF-2A electronic nose. Secondly, additional descriptors based on a coffee aroma kit such as Le Nez du Café, or the aroma wheel can be used to optimize the descriptions. Finally, the measurement conditions such as the sample volume, temperature, and gas dilution require optimization.

In this study, we showed that the FF-2A electronic nose that trained by the wine aroma kit indicated: (1) the differences among coffees; (2) the difference between drip coffee and canned coffee; and (3) the differences among coffee, green tea, and wine, based on their calculated similarities. The employed combination of the electronic nose and aroma kit can be used to describe aromatic features in an easily understandable way, and promise to bridge the gap between electronic noses and human olfaction.

## 4. Conclusions/Outlook

An electronic nose trained by the Le Nez du Vin wine aroma kit was successfully used to describe and distinguish among different coffee aromas. The method promises to be useful for the objective description of aromatic features in an understandable way.

## Figures and Tables

**Table 1.** Sample details.

**Sample**	**Type**	**Volume (μL)**
Drip 1	Medium roast coffee (Colombia)	200
Drip 2	Dark roast coffee (Multi-region)	200
Drip 3	Dark roast coffee (Multi-region)	200
Canned 1	Coffee with milk, sugar, & flavor	200
Canned 2	Coffee with milk, sugar, & flavor	200
Canned 3	Black coffee	200
Green Tea	Japanese green tea in PET bottle	500
Red wine	Cabernet Sauvignon & Syrah in Pack	5

**Table 2.** Volume and dilution ratios of flavor samples.

**Sample Name**	**Volume in 1st Bag (μL)**	**2nd Dilution Ratio**	**Sample Name**	**Volume in 1st Bag (μL)**	**2nd Dilution Ratio**
**1. Abricot**	5	×12.5	**27. Girofle**	5	×16.7
**2. Acacia**	5	×12.5	**28. Goudron**	20	×1
**3. Amande amere**	5	×12.5	**29. Iode**	5	×10
**4. Ananas**	5	×10	**30. Muscat**	5	×100
**5. Anis**	5	×12.5	**31. Menthe**	5	×25
**6. Aubepine**	5	×12.5	**32. Mercaptan**	20	×1
**7. Banane**	10	×25	**33. Miel**	5	×10
**8. Beurre**	5	×12.5	**34. Musc**	5	×20
**9. Cacao**	5	×12.5	**35. Noisette**	5	×1
**10. Cannelle**	5	×12.5	**36. Noix**	5	×25
**11. Café**	20	×1	**37. Orange**	20	×5
**12. Caramel**	5	×1	**38. Pivoine**	5	×16.7
**13. Cassis**	5	×10	**39. Pin**	5	×12.5
**14. Cerise**	5	×33.3	**40. Poire**	5	×16.7
**15. Champignon**	5	×25	**41. Poivre**	5	×25
**16. Chene**	5	×10	**42. Poivron-vert**	5	×10
**17. Citron**	5	×20	**43. Pomme**	5	×50
**18. Civette**	5	×12.5	**44. Prune**	5	×16.7
**19. Coing**	5	×12.5	**45. Rose**	5	×33.3
**20. Eglantine**	5	×33.3	**46. Soufre**	20	×1
**21. Foine coupe**	5	×33.3	**47. Thyme**	5	×20
**22. Fougere**	5	×50	**48. Tilleul**	5	×16.7
**23. Fraise**	5	×20	**49. Truffe**	5	×1
**24. Framboise**	5	×12.5	**50. Viollete**	5	×25
**25. Fume**	5	×50	**51. Vinaigre**	5	×1
**26. Geranium**	5	×16.7			

**Table 3.** Aromas recognized by the FF-2A electronic nose and their attribution in the wine and the coffee wheels.

**Aromas**	**Wine Aroma Wheel (Noble, A.C. *et al.* [16])**	**SCAA Flavor Wheel [17]**
**Coffee**	Burned, Woody	—
**Mushroom**	Earthy	Groundy
**Pine**	—	Turpeny, Resinous
**Honey**	Caramelized	Syrup-like, Caramelly
**Strawberry**	Berry, Fruity	—
**Mint**	Fresh, Vegetative	—
**Orange**	—	—
**Musk**	—	—
